# Novel concept of a modular hip implant could contribute to less implant failure in THA: a hypothesis

**DOI:** 10.1186/s13037-017-0148-7

**Published:** 2018-01-08

**Authors:** Ronny Grunert, Stefan Schleifenbaum, Robert Möbius, Michael Kopper, Christian Rotsch, Welf-Guntram Drossel, Niels Hammer, Torsten Prietzel

**Affiliations:** 1 Department of Orthopedic, Trauma and Plastic Surgery, University Hospital Leipzig, Liebigstrasse 20, 04103 Leipzig, Germany; 2 Department of Orthopedic and Trauma Surgery, HELIOS Clinic Blankenhain, Wirthstrasse 5, 99444 Blankenhain, Germany; 3 ZESBO – Zentrum zur Erforschung der Stuetz- und Bewegungsorgane, Semmelweisstrasse 14, 04103 Leipzig, Germany; 4 Department of Anatomy, University of Otago, Lindo Ferguson Building, 270 Great King St, Dunedin, 9016 New Zealand; 5 Institute of Anatomy, University of Leipzig, Liebigstrasse 13, 04103 Leipzig, Germany; 6 Fraunhofer institute for machine tools and forming technology, Nöthnitzer Straße 44, 01187 Dresden, Germany; 7 FTZ e.V. Westsächsische Hochschule Zwickau, Dr.-Friedrichs-Ring 2A, 08056 Zwickau, Germany

**Keywords:** THA, Modular femoral stem, Taper junction, Neck-stem interface

## Abstract

**Background:**

The modularity in total hip arthroplasty (THA) allows orthopaedic surgeons for an exact reconstruction of hip biomechanical parameters especially in revision and tumor arthroplasty. Modular structured femoral stems using taper junctions showed increased implant breakage in the recent past.

**Presentation of the hypothesis:**

We hypothesize that a novel modular stem-neck-interface leads to less implant breakage compared to conventional femoral stems.

**Testing of the hypothesis:**

For this purpose, a novel modular femoral stem for THA was to design and manufacture. Therefore, three different variants of interface mechanisms were developed that enable a simple connection between the stem and the neck modules and allow for intra-operatively adjustment. Three prototypes A, B and C were manufactured and subsequently dynamic fatigue (ISO 7206–6) and body donor tested.

**Implication of the hypothesis:**

Modularity in THA is mainly applied in THA as well as in revision and tumor arthroplasty. Modular implants are barely used because of the high risk of breakage. Another risks in this context are taper fretting, corrosion and disconnection. With the novel design, it should be possible to detach the stem and neck module intra-operatively to adapt the anatomical situation. The novel coupling mechanism of the rotating interface seems to be the most suitable for a secure stem-neck connection and is characterized by good intraoperative handling.

## Background

The most important treatment goal in total hip arthroplasty (THA) is the recovery of normal function. Key requirements for this goal include the permanent and secure anchoring of the implants used and an exact biomechanical reconstruction of patient anatomy. Some hip biomechanical parameters need to be adjusted according to the individual situation intra-operatively. These includes the position of the prosthetic femoral head centre in relation to the femoral bone as well as the position of the prosthetic acetabular cup centre in the pelvic bone. The immediate consequence of malpositioning is a leg length discrepancy, in the majority of cases an increase in leg length, noticeable by patient and requiring orthopaedic treatment [1].

Also relevant are changes to femoral offset. Offset reduction reduces the lever arm of the hip muscles and thus reduces their force. Both increases in leg length and offset reduction can be sensed by patients, reduces mobility and require permanent post-operative orthopaedic compensation (e.g. shoe elevation). Further treatment is often required, yet does not always lead to full compensation, often leaving patient’s with a limp or a Trendelenburg gait. Excessive changes in the biomechanics pose further risks, including e.g. dislocations, implant loosening, breakage and wear, potentially inducing wear-associated osteolysis [2, 3]. All these mentioned factors can lead to drastic cost increases due to new medication needs, physiotherapy and further orthopaedic treatment. To avoid such situations, the biomechanical aspects of the operation need to be looked at precisely during the operation.

One approach for the reconstruction of the original anatomical situation and biomechanics in primary THA is the deployment of a modular structure of the femoral stem. There are different solutions available such as the Metha-Stem of Aesculap (Aesculap, Tuttlingen, Germany) or the Oval-Stem of Zimmer (Zimmer, Warsaw, USA). Both approaches showed increased implant breakage and did not lead to the success hoped for [4–14]. One reason for this is the use of taper junctions between the stem and neck modules. The problem is that positioning the coupling module within the femoral medullary space requires a very fine implant design. The combination of taper interface and the long lever lead to a high peak strain that increases the risk of breakage in both components. The taper junction is also not able to absorb horizontal forces. In addition, this kind of connection is difficult to disassemble intra-operatively in order to adjust it.

### Presentation of the hypothesis

We hypothesize that a novel design of modular stem-neck-interface leads to less implant breakage compared to conventional femoral stems using taper connections. Furthermore, a simple intra-operatively handling for the surgeon should be ensured, which would help to select components with the ideal geometry to reconstruct the original hip architecture.

### Testing of the hypothesis

Using computer aided design (CAD), three different variants (A, B, C) of novel connection mechanisms were developed that enable a simple connection between the stem and the neck modules and allow for detachment and precise biomechanical adjustment intra-operatively.

#### Variant A: Sliding interface (conical dovetail)

**Fig. 1:**
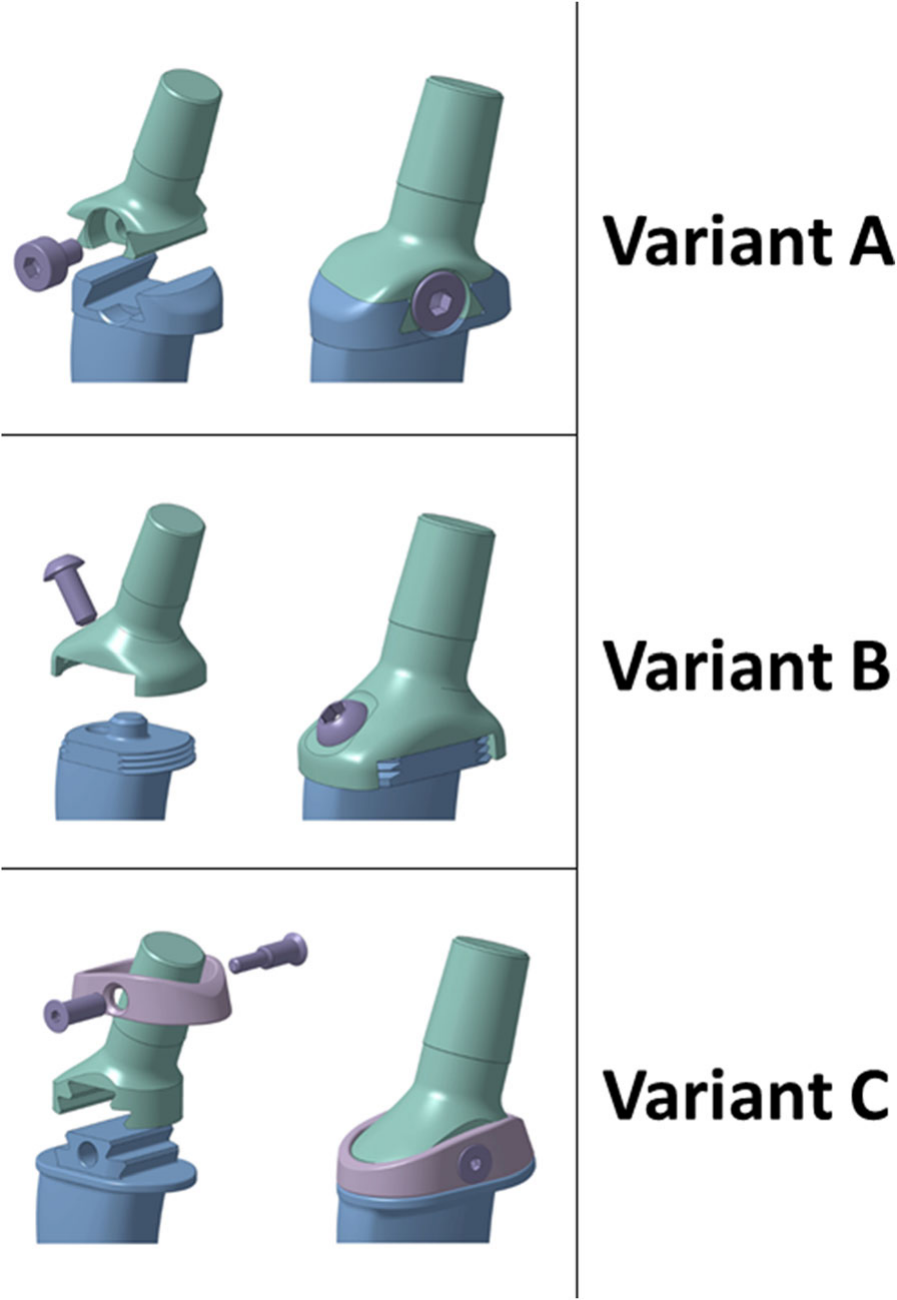
Concept variants for novel interface: **a** sliding conical dovetail interface, **b** rotating interface, **c** sliding double-lapped dovetail interface

This interface is a conical dovetail (Fig. 1). The neck component can be easily inserted and slid into the conical position to the mechanical end stop. The system is secured by a screw that is inserted into the dovetail and presses against the neck module to hold this component in the end position. The interface is also tilted diagonally to ensure that in case of loosening, the neck module will tend to move back into the conical position under strain.

#### Variant B: Rotatable interface (screw thread principle)

The head neck module is positioned at a 90° tilt onto the stem-module and centred above by means of a bolt. The neck module rotates around the central bolt, pulling towards the stem module via the screw thread and finally tightening against the larger flat surface. The system is stabilized with pegs, inserted during the operation (e.g. high-strength synthetic pegs). The interface itself serves as the collar of the prosthesis due to its protruding design (Fig. 1).

#### Variant C: Sliding interface (double-lapped dovetail)

The neck module is slid onto the stem module linearly. The interface is produced by a linear double-lapped dovetail. The system is sealed by means of a cap with a conical position and a self-locking sleeved screw through the interface. This interface variant can be made with and without a collar (Fig. 1).

#### Manufacturing the prototypes

**Fig. 2:**
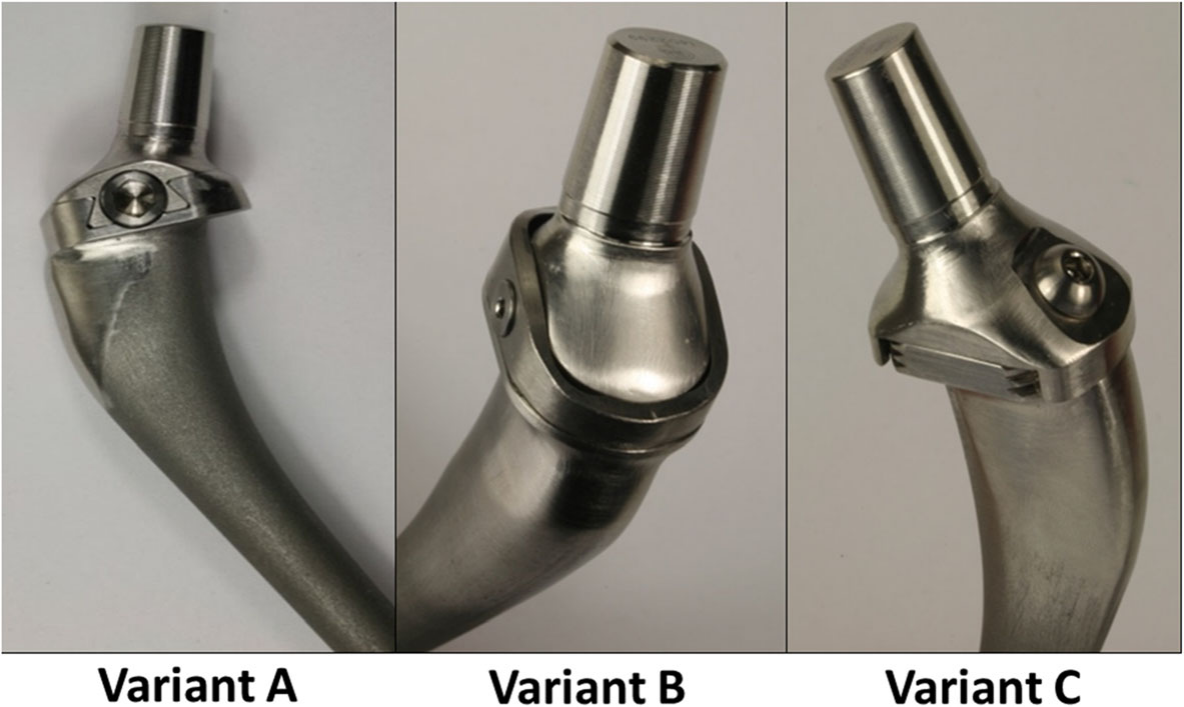
Prototypes for each variant: **a** conical dovetail, **b** rotating interface, **c** double-lapped dovetail

A functional prototype of each coupling mechanism was manufactured of the original alloy CoCr28Mo6 (Fig. 2). For this, original parts of the implant type Co Plan K size five (AQ Implants GmbH, Ahrensburg, Germany) were used. Processing of the cone was completed on the Chiron Mill 800 center. The unprocessed raw parts were separated above the resection plane and the stem modules were subsequently manufactured. Special moulds needed to be made for this. The semi-finished products were then again separated under the resection plane and the neck modules were manufactured from the remaining upper parts. A special clamping unit needed to be made to hold the neck components at the processed cone.

#### Dynamic fatigue limit testing

**Fig. 3:**
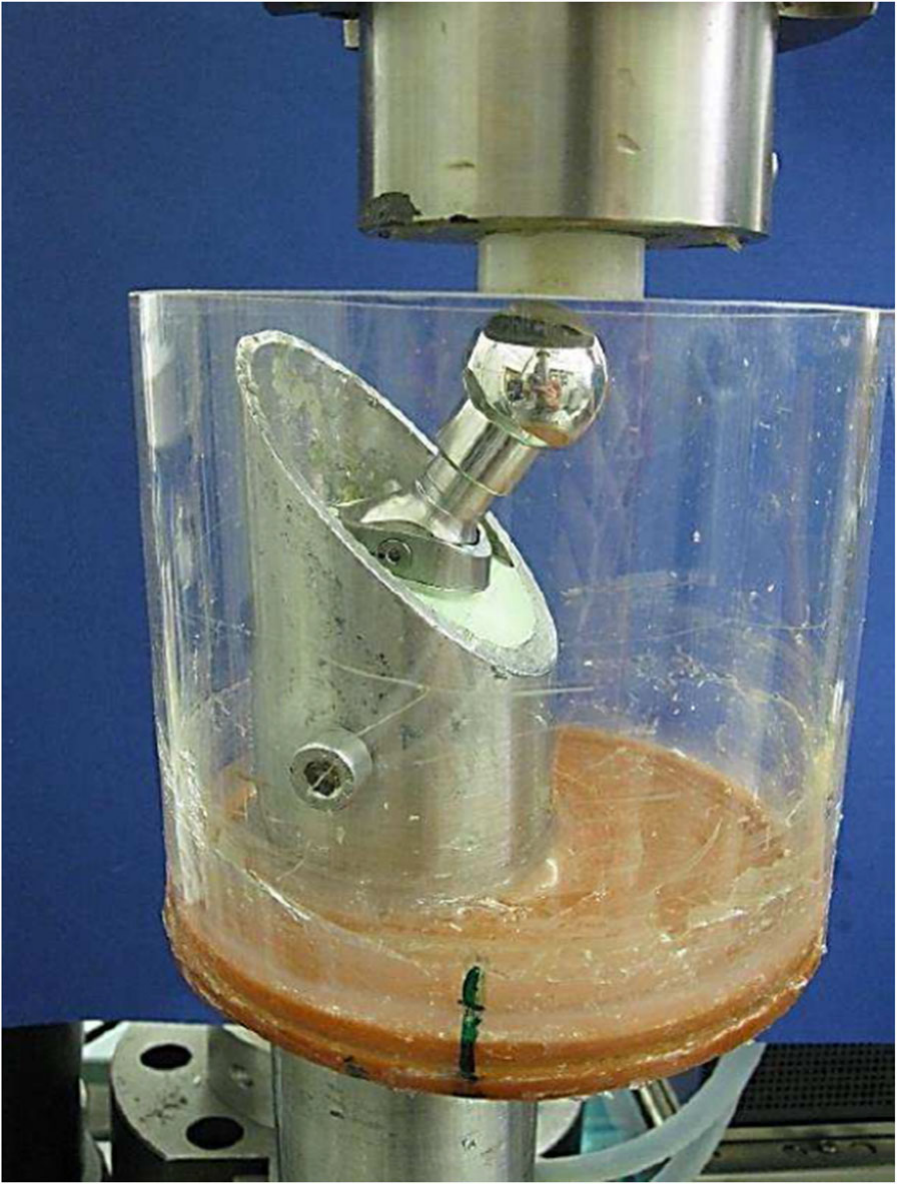
Experimental setup for dynamic fatigue limit testing according to the ISO 7206–6 standard

According to the ISO 7206–6 standard, fatigue limit was tested by a certified laboratory (IMA Material und Anwendungstechnik GmbH, Dresden, Germany) (Fig. 3). Testing was completed with a maximum force of 5340 N and at 10,000,000 cycles. The specimen was embedded in 0.9% NaCl solution.

#### Variant A - conical dovetail

**Fig. 4:**
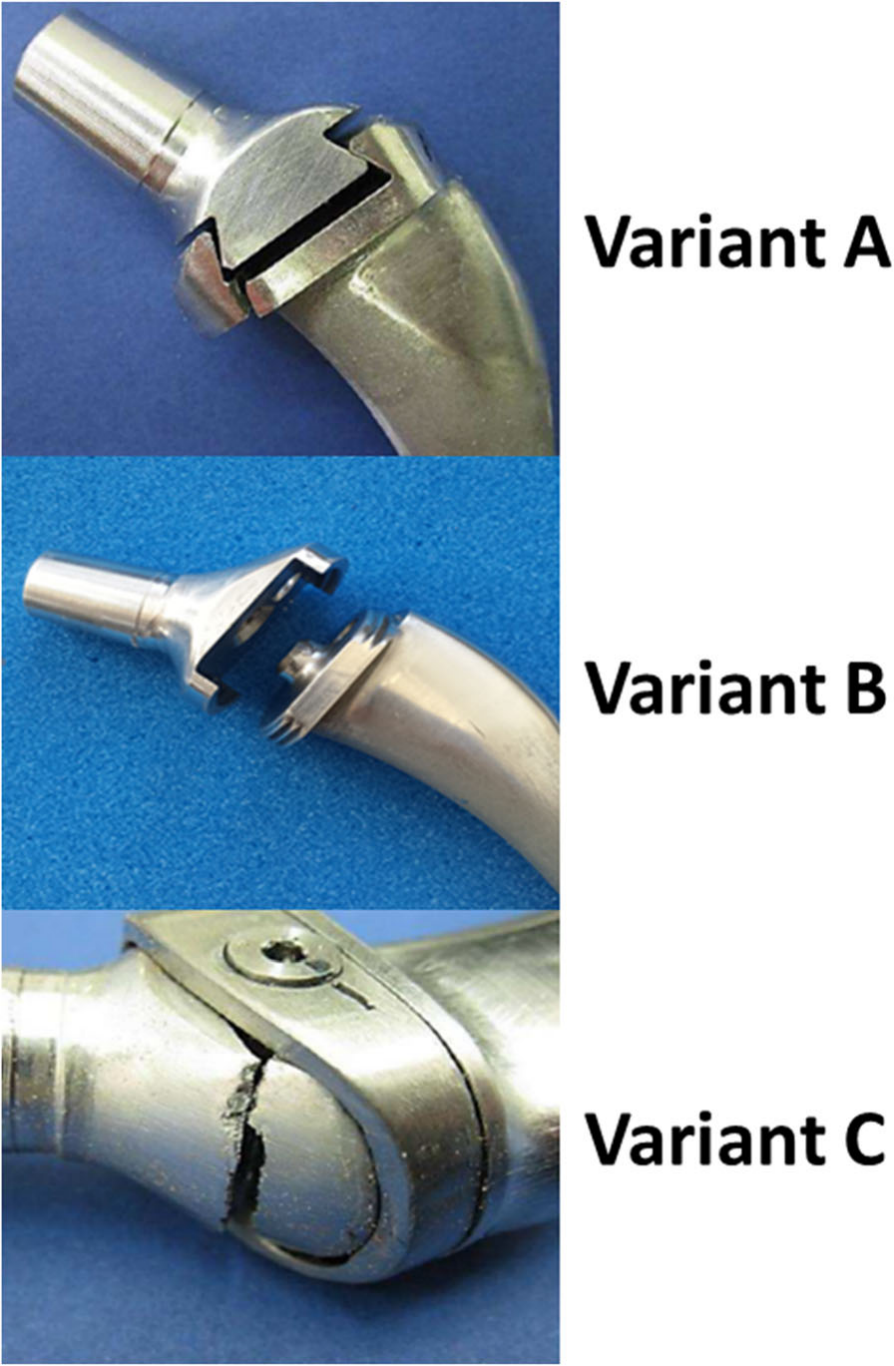
Failure pattern after fatigue testing: **a** breakage, **b** no damage, **c** breakage

As can be seen in Fig. 4, a crack has formed from the base of the dovetail, through the bottom up to the notch between the shaft and the interface surface. The loosening torque of the self-locking screw connection M5 was down to 20–25% of the tightening torque. In the resting state, the loosening torque consisted of 90% of the tightening torque. This does not necessarily have anything to do with the loosening of the screw connection. Following the strain, the conical dovetail connection is further driven into its position, which lowers the preload.

#### Variant B - rotating interface

After fatigue testing, no damage could be seen (Fig. 4). The loosening torque of the self-lock screw connection M5 consisted of 20–25% of the tightening torque. At rest, the loosening torque was 90% of the tightening torque. This is not necessarily related to loosening of the screw connection. As a consequence of dynamic strain, the rotating interface was ‘overwound’, which lowers the preload on the screw connection. This overwinding of 3° at the end of the experiment was clear and permanent.

#### Variant C - double-lapped dovetail

The horizontal crack occurred above the modular connection (Fig. 4). The loosening torque of the metric screw connection M3 was similar to the tightening torque. As a consequence of deformation and loosening of the connection, the preload was increased on the sealing cap due to the conical construction, without overloading the cap. This led to a perpendicular clamping effect on the screw connection.

### Implication of the hypothesis

Until now, modularity in THA is mainly applied in revision and tumor arthroplasty [15–18]. In primary THA modular implants are barely used because of the high risk of breakage [11, 19–21]. Another risks in this context are taper fretting, corrosion and disconnection which were observed [10, 12, 19, 22–25]. In contrast, some authors performed a simulation with in vivo conditions and recognized no corrosion increase at the interface [3]. Further, in primary THA the application of taper junction for connecting the implant modules leads due to the arrangement inside the femur to a filigree design (e.g. cone 10/12 mm) and unfavorable leverage ratios with peak loads in the medial force application. This results in high breakage rates in the case of the Metha-Stem or in the case of the oval handle [5–14]. In contrast for modular tumor endoprosthesis partially much larger taper connections can be applied (e.g. 16/18 mm). In this regard, a number of complications such as disconnections are reported, but no relevant breakage rates [16].

In the present hypothesis study, we introduced a new concept for a modular THA implant. With the novel interface design, it should be possible to detach the stem and neck module intra-operatively to adapt the anatomical situation. The use of a modular stem implant in primary arthroplasty would disconnect the neck in case of THA revision for a less invasive access to the cup or insert. Therefor the stem module remains in the femur during adjustment. New geometries for the neck module should be offered to the surgeon in order to adjust hip biomechanic parameters like leg length, femoral offset as well as the femoral anteversion [11–14] more flexible and precisely. The coupling mechanism of the rotating interface seems to be the most suitable for a secure connection. However, these findings are based on one sample of each coupling mechanism and do not represent a statistically verified test result. Thus, further development is necessary to optimize this coupling interface. This includes strengthening the wall-thickness to further decrease fracturing and increasing the notch radius at the interface to prevent cracking. Furthermore, increasing processing precision and increasing tightening torque for the rotating interface is required to prevent overwinding that could decrease preload.

## Conclusions

A newly designed femoral component, consisting of stem and neck module, was introduced. The main focus was on a better adaptability for the intraoperatively adjustment of biomechanical hip parameters e.g. the leg length as well as an increased resistance to implant breakage. With the novel design, it should be possible to adapt the anatomical architecture by using different combinations of stem and neck modules.

Variant B with the coupling mechanism of the rotating interface seems to be the most suitable for a secure connection when looking at the results of fatigue testing and intra-operatively handling. It will be examined for further testing, design guidance, simulation control and construction revisions. Thus, the results of this hypothesis study is intended as a basis for discussion for a novel thought to the development of hip replacements.
